# [Retracted] Nicotinamide N‑methyltransferase promotes epithelial‑mesenchymal transition in gastric cancer cells by activating transforming growth factor‑β1 expression

**DOI:** 10.3892/ol.2024.14475

**Published:** 2024-05-29

**Authors:** Liang Liang, Ming Zeng, Haixia Pan, Hao Liu, Yangke He

Oncol Lett 15: 4592–4598, 2018; DOI: 10.3892/ol.2018.7885

Following the publication of this paper, it was drawn to the Editor's attention by a concerned reader that the western blotting data shown in Figs. 1, 2B, 3A and B, and 4A, the scratch-wound assay data in Fig. 2C, and the migration and invasion assay data in Fig. 2D were strikingly similar to data that had either already appeared in previously published articles written by different authors at different research institutes, or were featured in articles that were submitted for publication at around the same time. In addition, two data panels were found to be overlapping in the cell morphological images shown in Fig. 4B on p. 4596, such that data which were intended to show the results from differently performed experiments were apparently derived from the same original source.

Owing to the fact that the contentious data in the above article had already been published prior to its submission to *Oncology Letters*, the Editor has decided that this paper should be retracted from the Journal. After having been in contact with the authors, they accepted the decision to retract the paper. The Editor apologizes to the readership for any inconvenience caused.

